# Automating tasks in protein structure determination with the clipper python module

**DOI:** 10.1002/pro.3299

**Published:** 2017-11-06

**Authors:** Stuart McNicholas, Tristan Croll, Tom Burnley, Colin M. Palmer, Soon Wen Hoh, Huw T. Jenkins, Eleanor Dodson, Kevin Cowtan, Jon Agirre

**Affiliations:** ^1^ Department of Chemistry, York Structural Biology Laboratory The University of York York YO10 5DD United Kingdom; ^2^ Department of Haematology, Cambridge Institute for Medical Research University of Cambridge Cambridge CB2 0XY United Kingdom; ^3^ STFC Rutherford Appleton Laboratory OX11 0QX Collaborative Computational Project for Electron cryo‐Microscopy (CCP‐EM) United Kingdom

**Keywords:** automation, pipelines, scripting, Clipper, libraries, Python, SWIG, crystallography, electron cryo‐microscopy, NumPy

## Abstract

Scripting programming languages provide the fastest means of prototyping complex functionality. Those with a syntax and grammar resembling human language also greatly enhance the maintainability of the produced source code. Furthermore, the combination of a powerful, machine‐independent scripting language with binary libraries tailored for each computer architecture allows programs to break free from the tight boundaries of efficiency traditionally associated with scripts. In the present work, we describe how an efficient C++ crystallographic library such as Clipper can be wrapped, adapted and generalized for use in both crystallographic and electron cryo‐microscopy applications, scripted with the Python language. We shall also place an emphasis on best practices in automation, illustrating how this can be achieved with this new Python module.

**Abbreviations and symbols (glossary)**
CCP4: A UK‐wide collaborative consortium devoted to collecting, supporting and distributing a suite of programs for macromolecular crystallography
CCP‐EM: Similar initiative to CCP4, but for supporting electron cryo‐microscopy methods
Clipper: An efficient C++ crystallographic library, written by Kevin Cowtan (University of York, UK)
CCP4i2: The new graphical interface to the CCP4 suite of programs, written in Python and heavily oriented towards pipelines
Fragon: A new pipeline for ab initio phasing from ideal fragments, distributed by CCP4
Crank‐2: A new pipeline for experimental phasing, optimized for structure solution from datasets containing weak anomalous signal
MRC file: A map format traditionally used for storing electron microscopy micrographs

## Introduction

The excitement that accompanies the interpretation of a new macromolecular structure is, at many levels, a well‐earned compensation for a prologue plagued with iterative, repetitive work: first the biochemical challenges (grow cells, purify, and try to crystallize a protein, sometimes for months or even years)—and then the structural ones (collect data, obtain electron density maps, build models, refine, and validate). At the latter stage, automation can offer two main functions. First, it can perform many uncomplicated tasks systematically and swiftly, and report success or failure clearly. An example is brute—force testing of many models for molecular replacement, then reporting R factors after preliminary refinement. Success is indicated by finding one trial that stands out as “better” from a cluster of other results. We will call these cases *exhaustive* solutions.

Second, decisions can be made to supplement the user's lack of expertise. Examples are: selecting the space group for a data set, choosing the resolution cut‐off for the experiment, choosing a hand after substructure detection based on how much of the structure can be built automatically, then proceeding to further model building. We will refer to these as *expert* solutions.

In certain scenarios, automation is more of a need than a commodity. Data acquisition in synchrotrons and XFEL facilities is nowadays so fast that user intervention in the processing and presentation of these data is neither required nor wanted, as this would slow the process down unnecessarily. Electron cryo‐microscopy (cryoEM) users may see that the third generation of direct‐electron detectors will correct beam‐induced particle drift transparently (done using GPGPU methods, General Purpose computing on Graphics Processing Units). Crystallographers at synchrotrons may be presented with datasets fully processed with the different algorithms embedded in state‐of‐the‐art software packages,^1^, ^2^, ^3^ together with relevant statistical quality indicators to inform the best choice of data for further analysis^4^, ^5^. Indeed crystallographic methods are now so mature that these data can now be fed into down‐line procedures such as phasing,^6^ selection of molecular replacement solutions followed by refinement,^7^, ^8^ or even preliminary model building^9^. It is quite possible that the user's involvement may begin with interpretation of features in a difference map.

The structure solution tools can also access excellent bioinformatics packages,^10^, ^11^, ^12^ which provide valuable supplementary information; for example, to select likely models, monomers, oligomers or domains to use as molecular replacement templates, to pinpoint likely N‐glycosylation sites, or to provide a high resolution structure that best fits into a moderate‐resolution cryoEM reconstruction.

Finally, linking these tools with validation and analysis software can allow self‐assessment capabilities, with expert scripts able to test their own hypotheses—for example, is the result satisfactory, as indicated by a simple statistic such as a low R‐factor or a high correlation coefficient for a sub‐structure solution.

Advances in scripting languages, both in terms of efficiency (byte code vs. fully interpreted code) and semantics (readable high level languages such as Python), have opened the door to new ways of automation. Modules such as lxml (for eXtensible Markup Language [XML] data) and json (for JavaScript Object Notation [JSON] object definitions) guarantee interoperability between different programs at the data exchange level. Furthermore, the integration of fully‐featured database systems such as SQLite into standard distributions of the Python language have also enabled the production of persistent pipelines: software pieces—either in binary form or as callable modules—glued together in such a way that data, state, results and all the decisions made are available to downstream, connected processes, offering the possibility of revising the whole process years later and by different people.

The development of pipelines within the Collaborative Computational Project No4 (CCP4^13^) and the Collaborative Computational Project for Electron cryo‐Microscopy (CCP‐EM^14^) has, at least until now, been limited by the wider availability of program libraries in compiled, machine‐dependent code. Aside from Coot,^15^ which is fully callable from both Python and Scheme languages, few other programs have offered scripting interfaces. However, the situation is rapidly changing. Recently, CCP‐EM has included the mrcfile library—a pure Python module for reading and writing MRC files—enabling developers to produce standard‐compliant map files in that format^14^. The introduction of the CCP4i2 interface, fully written in Python,^18^ has provided bespoke‐wrapped versions of many CCP4 programs, including Aimless,^16^ Buccaneer^17^ and Refmac5^18^. Although this opened up interesting possibilities for automation, there was still a major gap to be addressed within CCP4, which is the lack of a Python toolkit that links basic crystallographic operations to support the creation of novel scripted methods. The well documented, efficient Clipper C++ library^19^ provides precisely the required functionality.

Here, we show how a major pre‐existing C++ library such as Clipper can be conveniently wrapped with Python applications. Although the proposed method is not the only one available, it is the most convenient one when a pre‐existing C or C++ library is available.

## Wrapping a C++ Library for Use with Scripting Languages

For reasons of computational efficiency, most crystallographic libraries have been originally designed and implemented in compiled languages such as C and C++, but thanks to the wide availability of wrapper interface generators it is now possible to access them from scripting languages. Some popular interface generators are Simplified Wrapper Interface Generator (SWIG), Boost.Python and SIP. SWIG and Boost.Python are routinely used within crystallographic software suites.

Originally conceived to provide generic scripting capabilities, SWIG is able to translate classes, types and function calls, requiring only a textual interface definition from the developer. This interface file (see Fig. ,1 file “clipper.i”) can be reused to generate wrappers for other scripting languages, with little to no modification. From Python's perspective, some noticeable differences with the original C++ code include the use of a single namespace as opposed to multiple, nested ones in C++, and lack of support for templates. SWIG is widely used in the structural biology community, with programs such as Coot,^15^ CCP4mg,^20^ Privateer,^21^ CCP‐EM,^14^ and Modeller^22^ offering scripting capabilities through a wrapper interface generated with this software.

**Figure 1:**
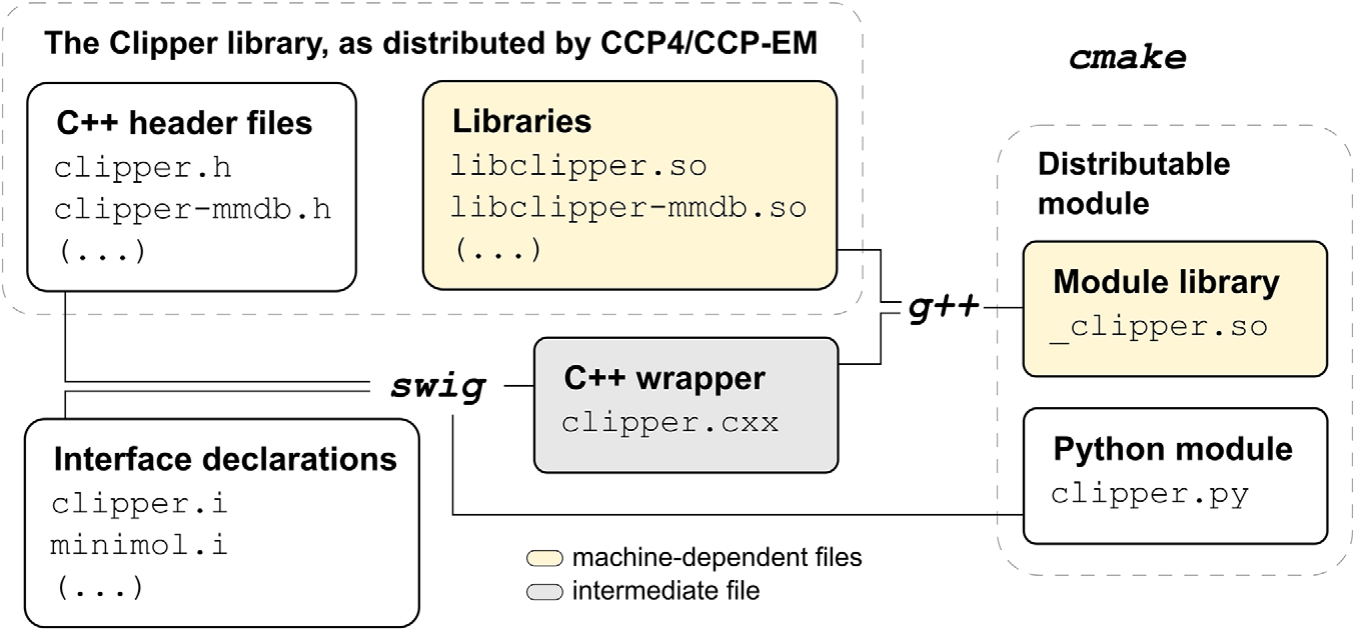
Overview of the files and commands involved in the generation of the Python interface. The build system chosen for this project is cmake, as it is well integrated with both CCP4 and CCP‐EM build pipelines. The only machine‐dependent files (highlighted in yellow) are Clipper's pre‐existing shared objects (.so) and the new shared object generated by SWIG as a companion to the Python interface. Both files (clipper.py and _clipper.so) are required for the module to work, and are distributed as part of CCP4 and CCP‐EM.

Boost.Python offers powerful bi‐directional interfacing capabilities between Python and C++, requiring only a C++ compiler and the Boost distribution to work. Boost.Python is most notably used within the crystallographic community as the main interfacing mechanism within the PHENIX project^23^ and the cctbx toolkit,^24^ allowing for the proportion of algorithms written in Python to rise up to 60% of the total distributed cctbx code without compromising interoperability (http://cctbx.sourceforge.net/current/tour.html).

Looking to the future, C++/Python bindings may be greatly simplified by taking advantage of C++11‐specific compilers. This allows lightweight bindings to be generated using pybind11 (https://github.com/pybind/pybind11), minimizing the need for extensive boilerplate code.

### A real world example: the clipper_python module

The Clipper C++ library,^19^ developed at the University of York and cornerstone to many different developments within the CCP4 suite (Buccaneer,^17^ Coot,^15^ Privateer,^21^ Aimless,^16^ and Blend^25^ among others), has been wrapped in the Python language using SWIG and made available with the standard CCP4 and CCP‐EM distributions, among others (please refer to the Availability section). The SWIG package was chosen in order to minimize issues with binary distribution in CCP4, as there are already a number of CCP4 programs that expose a Python scripting layer through SWIG, and the suite also maintains compatibility with pre‐C++11 systems—for example, Mac OS X 10.6. In the near future, the module will be made available through the Python Package Index under the name clipper_python. The clipper_python module is also now at the core of the CCP4 suite's new, Python‐based graphical interface (CCP4i2,^26^), and is being adapted in a joint collaboration between CCP4 and the CCP‐EM consortiums to hold big data structures such as the ones required by electron cryo‐microscopy. Clipper provides methods and classes that support all stages of the macromolecular structure determination process after data reduction, that is, it does not offer data structures for holding unmerged reflection data. Many of the most common tasks are implemented in optimized method‐classes, for example, calculation of structure factors with bulk solvent correction or computing σA‐weighted map coefficients.

In terms of design, the Clipper C++ library follows a number of guidelines that ease the many potential obstacles a C++ beginner programmer might encounter. These are, among others: support for either float or double, use of namespaces to avoid naming conflicts, avoidance of pointers in public APIs, the use of generic Standard Template Library (STL) containers instead of manually allocating and freeing memory, parameters which are passed by reference, and strict use of const. While all these guidelines make sense in a C++ context, their translation into a scripting language such as Python is not possible, as there are no pointers, templates are not supported, parameters are passed by assignment, and there is no notion of const.

Fortunately, SWIG provides many mechanisms tailored to dealing with these translation issues: instances of templatized data structures can be created—for example, name an xmap_float Python type for a clipper::xmap < float> C++ type ‐ to create a finite number of derived types. Also, as namespaces are flattened upon conversion, a %rename command is provided in order to resolve conflicts.

Adapting Clipper's C++ data structures to be accessed in Python's more natural way required extending the original classes with special accessor and modifier operations. For instance, __getitem__(index) and __setitem__(index, value) functions were implemented for most array classes under the %extend directive. Standard C++ list containers (std::vector<> in C++) are detected and automatically decorated with Python‐style functions by SWIG, making it possible to iterate over these list components in a manner familiar to the Python programmer. This is suitable for smaller lists (e.g., a list containing atomic contacts), but not for longer and unfortunately more common components (e.g., reflection lists or a succession of grid points in a map), where iterating over the elements would be too time‐consuming. Therefore, a decision was made to interface the bigger data containers to NumPy,^27^ which in addition to speeding most operations up and providing a wealth of optimized numerical functions, will also open the door to offloading certain calculations to GPU processors seamlessly in the near future^28^.

The need to carefully wrap data structures for fast and Pythonic access and modification extends to simpler data types as well. As an example, existing get and set functions for coordinates contained in the Coord_grid object appear in the C++ library as:

inline const int& u() const {return (*this)[0];}//!< get u

inline int& u() {return (*this)[0];}//!< set u

and similar for the v and w grid coordinates. While excellent C++ style, this presents a dual problem for wrapping in Python: (1) the identical naming of the get and set functions creates a naming conflict in SWIG; and (2): access from Python would require three (slow) Python function calls for every (u,v,w). These have therefore been renamed to hidden functions using the %rename directive, and replaced with Python‐friendly properties. Constructors and common mathematical functions have also been defined, allowing calls such as the following (where uvw is any Python iterable of three ints):

import clipper_python as clipper

uvw = [1, 2, 3]

this_coord = clipper.Coord_grid(uvw)

grid_vals = this_coord.uvw # returns a NumPy array of 3 ints

this_coord.uvw = uvw

new_coord = this_coord + [1,2,3] # returns a new Coord_grid object

Note that the latter method is only valid when the Coord_grid object is on the left hand side of the equation. Implementation of getter/setter functions as properties has been implemented for most classes exposed to Python.

**Figure 2:**
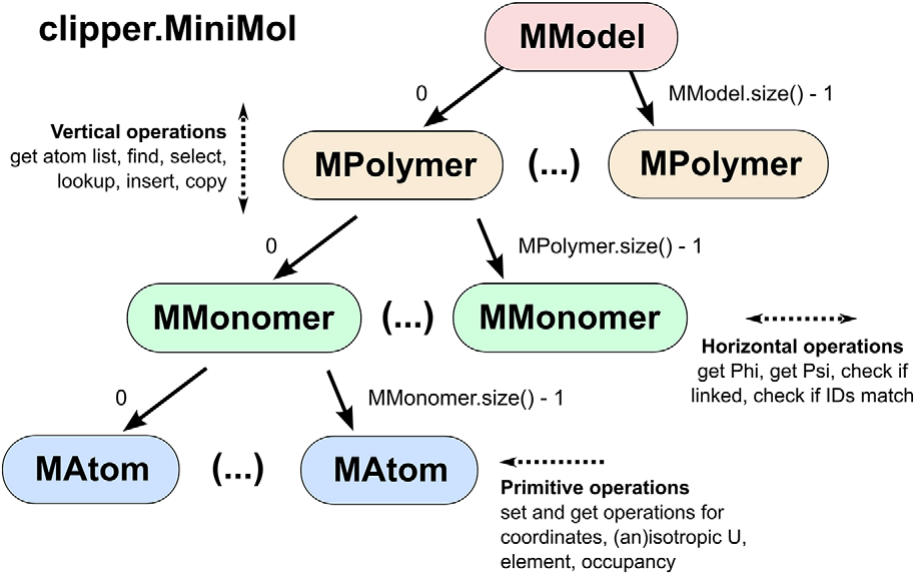
Structure and functions within clipper.MiniMol. The original C++ implementation of MiniMol makes use of STL vectors, which can contain MModel, MPolymer, MMonomer or MAtom. These have been extended to allow for natural Pythonic access (see example in the main text).

As an example of accessing Clipper's data structures in a Pythonic way, consider the following code, which reads a clipper.MiniMol structure (Fig. 2):

import clipper_python as clipper

f = clipper.MMDBfile()

f.read_file (“test.pdb”)

mmol = clipper.MiniMol ()

f.import_minimol (mmol)

for polymer in mmol.model ():

for monomer in polymer:

for atom in monomer:

print atom

This example shows that MModel, MPolymer and MMonomer are now iterable objects, while MAtom has been extended to include a __str__() function that collates the atom's ID with the output of MAtom.format() for convenience.

Similarly, a reflection list can be exported into NumPy and its amplitudes squared for the calculation of a Patterson function^29^:

numpy_data = hkl_data.export_numpy() # export NumPy array from HKL_data_F_Phi

numpy_data[:,1] = 0 # set phases to zero

numpy_data[:,0] = numpy_data[:,0]**2 # square the amplitudes

A companion module (clipper_tools) is being distributed with CCP4 and CCP‐EM alongside the clipper_python module, providing a growing set of pluggable components for automating the most common tasks in both X‐ray crystallography and cryoEM. These offer logging capabilities and create results in XML. As a further example, the ‘Analyse model geometry’ task in CCP4i2^26^ is the first program within this interface to take full advantage of the clipper_python module.

### Integration with pre‐existing packages

Pre‐existing structural biology packages (in particular those involved in molecular visualization) will already have their own data structures and methods for handling atomic coordinates, transformations *etc*., but may be enhanced by the use of the clipper_python module to provide handling of map coefficients and crystallographic symmetry. Thus, it is particularly vital that fast Pythonic methods are provided for the most common functions: interconversion of atomic coordinates between Clipper and host data structures; export of local map regions (e.g., to provide a live “box” of density that moves as the user pans through a structure); lookup of the necessary symmetry operators to pack a given region in space with copies of the atomic model; retrieval of the affine transformation matrices corresponding to a given set of symmetry operators.

***Interconversion of atomic coordinates***. The Atom_list class in Clipper is a simple wrapping of std::vector < clipper::Atom>. While this is a useful and efficient structure in C++, a straightforward wrapping into Python would require extensive use of (slow) Python loops for initialization and setting/accessing atom properties. Substantial extensions have therefore been added to this class to provide the necessary fast array functions, for example:

atoms = Atom_list(elements, coords, occupancies, u_isos, u_anisos, allow_unknown_atoms = False)

current_coords = atoms.coords # Returns a nx3 NumPy array

atoms.coords = new_coords # Takes any valid nx3 array of floats

atoms.u_anisos = u_anisos # Takes a nx6 NumPy array. For atoms with

# purely isotropic B‐factors, fill their

# row with isotropic values.

***Export of local map regions***. The Xmap and NXmap classes have been extended with fast functions to export map fragments to NumPy arrays. These class types differ in that the NXmap non‐crystallographic map class stores a map of arbitrary data type that is finite in extent and has no symmetry, whereas Xmap is specific to crystallographic maps, and contains the symmetry information required to appear infinite.

Options are provided to encompass most data organization strategies:

# Creates and fills a NumPy array encompassing all grid coordinates

# between start_coord_grid and end_coord_grid, in C‐style row‐major

# order and with the x axis first.

map_box = xmap.export_section_numpy(start_coord_grid, end_coord_grid,

order = 'C', rot = 'xyz')

# Re‐fills the existing NumPy array defined by map_box with data

# starting at start_coord_grid, in Fortran‐style column‐major

# order and with the Z axis first.

export_section_numpy(start_coord_grid, target = map_box, order = 'F', rot = 'zyx')

Note that the export_section_numpy methods export data on the same axes and with the same spacing as the data stored in Clipper (i.e., the crystallographic axes in the case of an Xmap). If data is required on strictly Cartesian axes this can be achieved using export_interpolated_box_numpy(), but this of course involves a large performance penalty.

***Lookup of symmetry operations***. A very common requirement when working with crystallographic data is to expand a model to include its symmetry neighbours, or to pack a given volume in space with symmetry‐equivalent molecules. This is another task that involves large loops which would become prohibitively slow if implemented in Python using directly‐wrapped Clipper functions. We have therefore created a new Unit_Cell class specific to the clipper_python module, such that:

#reference_coord: an (x,y,z) coordinate, typically the centroid of

# the atomic model.

#atom_list: the Clipper Atom_list object holding the asymmetric

# unit

#cell, symmetry, spacegroup, grid_sampling: standard Clipper objects uc = Unit_Cell(reference_coord, atom_list, cell, spacegroup, grid_sampling)

#all symmetry operators necessary to pack one unit cell starting

#from the model

symops = uc.symops

#all symops necessary to pack a given volume box_ops = uc.all_symops_in_box(origin_xyz, box_size_uvw, always_include_identity = False, sample_frequency = 2)

The chosen search algorithm trades off accuracy for speed, erring on the side of inclusiveness. In brief, a reference box is defined in grid coordinate space as the smallest parallelepiped encompassing the atomic model. The target volume is then searched in steps of the shorter of (shortest reference box side length)/(sample frequency) or the step to the next edge/face. For each search point, the inverse symmetry operator(s) mapping the point into the reference volume are determined. Finally, the symmetry operators found are sorted and culled to remove duplicates.

***Retrieval of affine transformation matrices***. Each symmetry operator consists of a 3 × 3 rotation matrix and a 3 × 1 translation vector. In order to aid fast retrieval into Python we have added functions to export these either individually or combined into a single NumPy array. Additionally, we have created a new Symops object (returned by the above Unit_cell methods). Using the box_ops example above:

# Returns an nx3x4 NumPy array providing all transformation matrices

# in the Symops object. The argument format='4x4' adds the row

# [0,0,0,1] to each matrix to create the full affine transformation

# matrix.

transforms = box_ops.all_matrices_orth

(cell, format='3x4')

An example of a working Clipper plugin to an existing visualization package (ChimeraX^30^) is available on request from the authors, or by installing the most recent ChimeraX build (https://www.rbvi.ucsf.edu/chimerax/download.html ) and downloading the plugin via its Toolshed (Tools/More Tools). Once installed, a crystal structure with live scrolling of maps and live display of local symmetry atoms can be instantiated by first loading the PDB or mmCIF file into ChimeraX, then opening the Python console (Tools/General/Shell) and typing:

from chimerax.clipper import CrystalStructure

m = session.models.list()[0]

cs = CrystalStructure(session, m, 'maps.mtz')

where maps.mtz contains at least one set of pre‐calculated map coefficients. Note that this plugin is still in development and experimental.

### Error handling

A danger when calling a C++ library from Python is that any unhandled C++ exception will crash the entire Python session. It is therefore very important to ensure that all possible exceptions are converted to Python objects to be handled by the calling code. Within the clipper_python module, we have chosen to map clipper::Message_fatal (C++) to RuntimeError (Python), std::out_of_range to IndexError, std::length_error and std::invalid_argument to ValueError and any other std::exception to UnknownError.

### Testing and documentation

As the documentation for the Clipper C++ library was originally produced using Doxygen (http://www.doxygen.org), the optimal way of producing documentation for the clipper_python module would involve channelling this information through the Python help system. Fortunately, this can be accomplished with the doxy2swig script (https://github.com/m7thon/doxy2swig ), which can automatically translate XML files—produced using a non‐default option in Doxygen—into SWIG interface files, which can then be included in from the main interface file.

Tests are currently being written using Python's unittest system. The module is automatically tested on a daily basis as part of the CCP‐EM Jenkins CI system (https://jenkins.io).

### A note on efficiency

**Figure 3:**
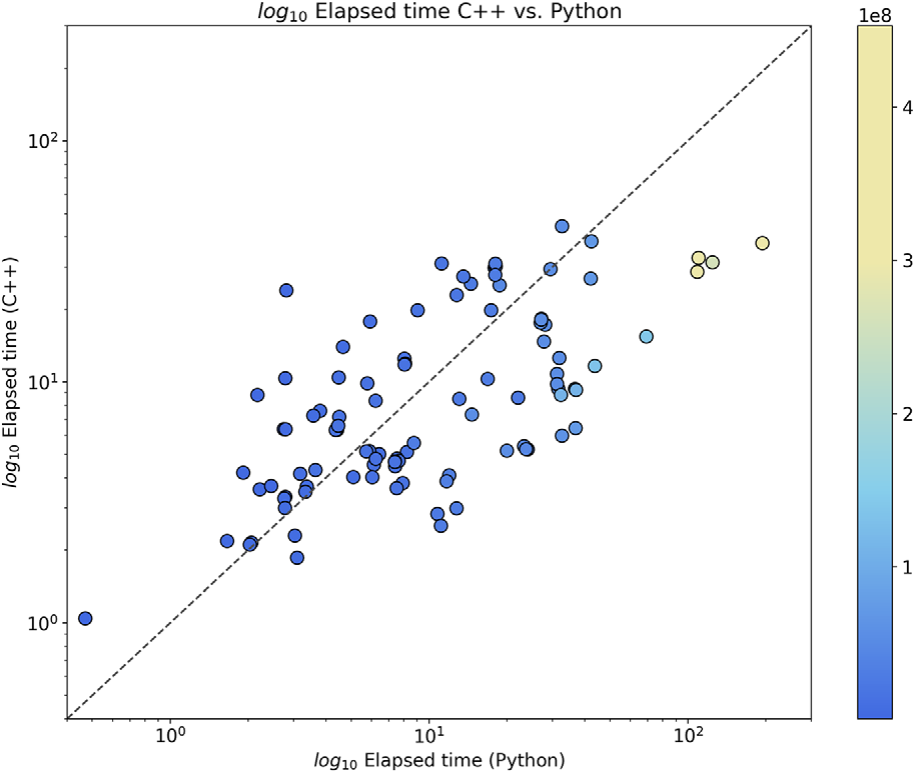
Comparison of C++ and Python run times. The run times of a map cutting tool written in C++ (cmapcut.cpp from CCP4, using the Clipper library) VS a Python counterpart (cut_by_density.py from the clipper_tools module, using the clipper_python module and NumPy). The test dataset was composed of 100 entries—map and fitted atomic model—obtained from the Electron Microscopy Data Bank (EMDB [19]) which were selected based on resolution—better than 4.0 Å. The results (elapsed time, measured using the time UNIX tool) were plotted with MatPlotLib [14], scaling both axes by log10.

As most of the computations are done in the original binary library, the clipper_python module does not exhibit the large performance penalty traditionally found in pure Python programs. Moreover, its interface to NumPy, itself a speed‐optimized general purpose numerical computing module, makes some tasks efficient and very straightforward in Python. As an example, a rough comparison is provided (Fig. 3) for two tools of equivalent functionality, implemented in C++ (Clipper) and Python (Clipper and NumPy). The programs—cmapcut from CCP4 and cut_by_density.py from the clipper_tools companion module—take a model and a map as input and output a map (or map coefficients, with an optional B‐factor correction for sharpening or blurring the transformed map). The results suggest that, for smaller map sizes (blue dots, plotted according to the colour bar on the right), running times are only marginally shorter or longer for either program, while bigger map sizes (light blue or yellow dots) clearly favor the C++ alternative. This may be due to the need to copy and relocate very large arrays in memory in the Python version—optimizations might be possible to alleviate this.

## Automation Through Scripting

In a computing context, a pipeline is a collection of programs arranged in a way that the output of each program serves as input for the next one. As mentioned before, pipelines mainly serve two purposes: combinatorial testing of options (exhaustive pipeline), and performing all tasks within a well‐defined process in an informed way, making choices along the way and reporting these at the end of the execution (expert pipeline). In the field of crystallography, some examples are MrBUMP^10^ (mainly exhaustive), Balbes^31^ (exhaustive), Arcimboldo^32^ and Arcimboldo_lite^12^ (exhaustive and expert), PDB_REDO^33^ (expert) and Crank2^9^ (expert). For the cryoEM field, pipelines in CCP‐EM^14^ include Refmac^18^ (expert), DockEM^34^ (exhaustive) and Flex‐EM^35^ (expert). It is also worthy of mention that, although a popular cryoEM reconstruction package such as Relion^36^ is not distributed as a pipeline, it has recently introduced the graphical creation of workflows, which closely resemble the functionality of a pre‐designed pipeline^37^.

### Structure of a pipeline

The code flow within a pipeline will differ significantly between the *exhaustive* and *expert* types. While the former will essentially consist of a main loop which will go through all the different possibilities to be tested—opening the door to using parallel job dispatchers such as GNU Parallel^38^—the latter will typically be organized in sequential blocks of code, tasks indeed, best organized in functions or modules and representing the logic of the pipeline—for example, auto‐build a protein model, refine it and only model waters if R‐factor goes below 0.30.

It is important to decide how best to communicate information between tasks. As certain scripting languages such as Python offer the possibility of returning multiple values, it is tempting to simply re‐use these variables as input for the next task; however, pipelines often run for much longer times than interactive tasks, making interruptions an event to consider. To this effect, persistent storage of intermediate results—that is, writing them to disk—provides a way of continuing interrupted jobs. Basic crystallographic data structures—for example, maps, models or reflections—can be dumped to disk in standardized formats—CCP4/MRC map format, PDB file format or MTZ file format respectively for the above examples—but writing other, non‐standard results usually requires the choice of a structured textual representation—for example, XML or JSON files, although a popular toolkit such as cctbx provides a module for handling its own file format (Python‐based hierarchical Interchange Library), which deals with task parameter communication.

### The choice of standard for communication files and data structures

The most popular available options, XML and JSON, were originally created to answer different needs. The XML was created as a flexible text format that could serve as the basis for markup languages such as HTML. Some examples of binary programs in the macromolecular crystallographic environment capable of XML output are Aimless,^16^ Privateer,^21^ and ARP/wARP^39^. XML is a typically verbose way of representing results, with a requirement to write opening and closing tags for each piece of representable data instead of a one‐off definition in the header, followed by structured data. JSON is also a structured data format, but it was designed for a different purpose: to provide a human‐readable way of transmitting data objects. The JSON format allows for more compact data strings, in a programming language‐like structure. Both formats are flexible enough for representing textual data, and both have libraries available for reading and writing results from major scripting languages. As real‐world examples, both the Protein Data Bank in Europe and CCP‐EM represent their structural data objects in JSON internally, while the choice within CCP4i2 and clipper_tools has been XML^26^.

As an example of a structured‐data report in XML:

<program name=“cut_by_model” user=“jon” date=“Fri Feb 24 11:14:24 2017” ok=“yes”>

<parameters mapin=“././unittests/test_data/emd_5148.mrc” pdbin=“././unittests/test_data/emd_5148_3ktt.pdb” b_factor=“−10.0” resolution=“2.5” mask_radius=“2.5”/>

<input_file name=“././unittests/test_ data/emd_5148 _3ktt.pdb” type=“PDB” ok= “yes”/>

<input_file name=“././unittests/test_ data/emd_5148 .mrc” type=“xmap” ok=“yes”/>

<output_file name=“mapout_cut_density. mtz” type=“mini MTZ” ok=“yes”/>

</program>

The same report in JSON format would look like this:

{

“program”: {

“‐name”: “cut_by_model”,

“‐user”: “jon”,

    “‐date”: “Fri Feb 24 11:14:24 2017”,

“‐ok”: “yes”,

“parameters”: {

“‐mapin”: “././unittests/test_ data/emd_5148.mrc”,

“‐pdbin”: “././unittests/test_ data/emd_5148_3ktt.pdb”,

“‐b_factor”: “−10.0”,

“‐resolution”: “2.5”,

“‐mask_radius”: “2.5”

},

“input_file”: [

{

“‐name”: “././unittests/test_ data/emd_5148_3ktt.pdb”,

“‐type”: “PDB”,

“‐ok”: “yes”

},

{

“‐name”: “././unittests/test_ data/emd_5148.mrc”,

“‐type”: “xmap”,

“‐ok”: “yes”

}

],

“output_file”: {

“‐name”: “mapout_cut_density. mtz”,

“‐type”: “mini MTZ”,

“‐ok”: “yes”

}

}

}

As automatic conversion between the two formats is entirely possible in Python, choosing one over the other is not likely to mark a point of no return.

A note on format interoperability: within clipper_python, it is possible to read and write most universally used formats—for example, PDB, mmCIF, MTZ, CCP4 maps. Other formats, such as those composed using plain text, must be read and converted using the Python language's standard facilities for reading and writing files.

### Persistence

Although most persistence requirements can be fulfilled simply by using structured results files, a popular option for indexing (and serving) results quickly is using a database engine. Python incorporates a module for accessing SQLite, which offers a complete single‐user database system that can be accessed with great simplicity, and is efficient across many configurations except perhaps those using network or distributed file systems. Alternatively, Python provides bindings to MongoDB, a NoSQL alternative to SQLite which uses JSON intermediates.

### Results presentation

Python provides graphical facilities for the creation of ad‐hoc reports (https://wiki.python.org/moin/NumericAndScientific/Plotting), with modules such as MatPlotLib^40^ being a popular choice for graphs. Within the CCP4^13^ and CCP‐EM^14^ suites, the JSrview framework provides an excellent alternative for real‐time reporting in Python, as its *pyrvapi* bindings provide function calls for the creation of HTML5/Javascript reports. In addition to several programs in the aforementioned suites, this package is successfully being used by many pipelines hosted by CCP4‐online. If reports are not to be generated using live calls to an API, they can also be created by a separate module which should read intermediate results (XML or JSON files, for instance) and produce a summary of the execution. This approach is exemplified by the CCP4i2 report mechanism,^26^ which was specifically designed to cope with parsing log files from legacy binary applications unable to be updated to produce structured data formats.

Exhaustive pipelines require a streamlined presentation, as the potential sheer number of results can impair the user's decision‐making process. Examples of this are Fragon^41^ or MrBUMP^10^. Expert pipelines on the other hand should expose their decision making process clearly, allowing the user to inspect the choices and, potentially, making it easy to tweak these in subsequent runs. This is particularly well exemplified by the Crank‐2 pipeline^9^.

## Conclusions

In this work, we have demonstrated how an existing C++ library can be adapted to work with a scripting language such as Python. Moreover, its combination with the NumPy module has provided a convenient way around traditional Python speed bottlenecks. Finally, the recent introduction of the CCP4i2 and CCP‐EM Python graphical interfaces coupled with the wide availability of free complementary Python modules (e.g., databases, structured data formats, access to various network protocols or code offloading to massively parallel architectures) will turn both CCP4 and CCP‐EM into fertile ground for automation.

## Availability

The clipper_python module is free software and it has been released under the terms of the GNU Lesser Public Licence (LGPL v3). There are several ways of obtaining it: through CCP4 or CCP‐EM (either as binary distributions or as source code), as a ChimeraX plugin and, in the near future, as a regular Python package installable by issuing the pip install clipper_python command. Also, sources are available through GitHub (https://github.com/clipper-python).
